# Practical tips for teaching the undifferentiated medical student in the emergency department

**DOI:** 10.12688/mep.19776.1

**Published:** 2023-10-18

**Authors:** Allan D. Winger, Dimitrios Papanagnou

**Affiliations:** 1 Department of Emergency Medicine, Thomas Jefferson University, Philadelphia, Pennsylvania, 19107, USA

**Keywords:** emergency medicine, clerkships, undergraduate medical education, clinical learning environment

## Abstract

Emergency medicine clerkships have become more prevalent in the third year of medical school, a time when students are immersed in the core clinical training of their undergraduate medical education. There is little guidance for clinician educators, however, on how to effectively scaffold learning for third-year medical students when rotating in the emergency department (ED) during core clerkships. The authors sought to provide best practices in teaching to leverage the rich learning environment of the ED – regardless of their specialty selections. Based on an extensive review of the literature spanning on-shift teaching, feedback, clinical medicine, and bedside teaching, the following twelve tips are offered to guide the instruction of the undifferentiated third-year medical student in the ED.

## Introduction

The paradigm shift of moving emergency medicine (EM) clerkships into the third year of medical school entails that medical, not medial students may require more support and on-shift teaching ( Tews *et al*., 2011; Tews *et al*., 2015) as they navigate their decision to pursue a specific specialty. Much has been written about developing a curriculum for third-year medical students during their EM clerkship ( Guth *et al*., 2020; Tews *et al*., 2011; Tews *et al*., 2015), as their learning goals are inherently different from those of their fourth-year counterparts. Unfortunately, few guidelines currently exist to aid clinician educators in tailoring their instruction for students rotating through the emergency department (ED) who are undecided on their future specialty and preparing them as physicians in the broader sense. We define this student as the undifferentiated medical student with regards to medical specialty selection and career planning. We share 12 practical tips for EM educators to fully leverage the entire breadth of educational opportunities the ED offers to maximize student learning in the context of uncertainty regarding future specialization. The first eight tips describe recommendations that are directed towards the individual learner, while the latter four describe interventions that will require support from departmental and educational leadership.

## Tip 1: Tailor student and educator expectations

The learning objectives for third-year and fourth-year EM clerkships are fundamentally different. Third-year learners are tasked with recognizing the structure and function of the ED, focusing on basic principles of EM and the approach to managing the undifferentiated patient ( Tews *et al*., 2015), as well as developing their basic history and physical examination skills ( Coates, 2004). By contrast, more “seasoned” fourth-year students have experienced an entire year of “core” clerkships during which they have practiced their history-taking and examination skills, and are primarily focused on rapid and efficient patient evaluation ( Tews *et al*., 2011), as well as developing higher-level diagnostic and management plans ( Tews *et al*., 2015). EM educators should, therefore, recognize that the clinical acumen of third-year students will necessarily be different than that of fourth-year students, and tailor expectations accordingly. Clinical acumen will also differ depending on the time of year, as students later in the academic year will have had more clinical experience outside of the ED ( Tews *et al*., 2015). Explicitly establishing expectations of both learner and educator at the outset can facilitate learner-centered, authentic teaching, encourage bi-directional feedback ( Natesan *et al*., 2023), and foster a stronger teacher-learner relationship.

Cognitive load theory also reminds us that the human brain can only process so much information at any given time and that “information overload” may impair knowledge retention and learning ( Natesan *et al*., 2020). Consequently, educators may consider affording more on-shift time to their students to process data and reduce any cognitive load associated with this unique clinical learning environment. Due to limited clinical experience, novice learners may require additional time to process and develop their clinical knowledge base. Allowing them to digest data, activate their existing fund of knowledge, and synthesize information from the patient history and examination have the potential to maximize the value obtained from each patient encounter ( Rencic, 2011).

## Tip 2: Diagnose the learner, not just the patient

Rotating third-year students have a paucity of clinical experience, particularly those for whom EM is their first clinical rotation. It is critical for the educator to intentionally consider content for teaching. Taking a moment to determine the learner’s objectives at the start of a shift can yield more relevant teaching moments ( Chinai *et al*., 2018), and provides an opportunity for more directed feedback at the end of shift. Asking probing questions to assess the understanding of a disease process, diagnostic test, or treatment modality may uncover knowledge gaps and identify opportunities for self-directed learning outside of the clinical setting ( Rencic, 2011). A tried-and-true teaching modality, the Socratic method of asking questions such as “why?”, “how would you approach scenario X?”, or “what if Y was different, how would you alter your plan?” can assist in uncovering knowledge gaps and provide opportunities for teaching and/or post-shift reflection ( Natesan *et al*., 2020). This has the benefit of guiding the learner out of the basic recall of facts and towards critical thinking.

 Because of the high volume and variable acuity of patients who present to the ED, however, it may not always be possible to engage in in-depth discussions or probe a learner’s knowledge base with a series of questions. An unusually busy shift or the number and/or complexity of resuscitations, for example, may limit the amount of time an educator can realistically spend discussing cases with a learner ( Chaou *et al*., 2019). In these instances, it is important for the educator to 1) anticipate learner uncertainty regarding a clinical situation in order to recognize a potential learning opportunity ( Chaou *et al*., 2019); 2) select the most appropriate pearl according to the learner’s self-identified objectives; and 3) provide targeted feedback that addresses the learner’s goals and behaviors. Attention should be taken to avoid targeting feedback towards a learner’s judgement, as this has the potential for being perceived as an attack on their personality, which can impact the rapport between educator and learner ( Natesan *et al*., 2023).

## Tip 3: Maximizing the student role

Much has been written describing the challenges of teaching in the ED. Barriers to teaching include limited time and frequent interruptions ( Chinai *et al*., 2018; Guth *et al*., 2020), unpredictable clinical conditions ( Chaou *et al*., 2019; Natesan *et al*., 2020), and the perceived trade-off between high-quality patient care and meaningful clinical instruction ( Chaou *et al*., 2017; Chinai *et al*., 2018; Colletti *et al*., 2012; Guth *et al*., 2020). In fact, one study examining perceptions of feedback in the ED found that 76% of attendings reported forgetting to give their residents feedback, and “being busy” was cited as the top influencing factor when providing feedback ( Chaou *et al*., 2019). In the fast-paced and often chaotic setting of the ED, a potential pitfall is assigning the learner tasks that help the workflow of the clinician educator. While collaboration can be beneficial to patient, learner, and teacher, it is important to avoid limiting the student’s decision-making role, as this can have the effect of likening them to a scribe ( Coates, 2004).

Instead of the aforementioned, valuable student contributions include taking a history, performing a physical exam, obtaining collateral information, and documenting their thought process in the form of notes. Because their patient load will necessarily be less than that of the educator, keeping abreast of clinical developments and informing the educator with what they think the next step should be is both helpful from a workflow standpoint and integral to the learner’s sense of ownership of their patient’s care. All of these actions simultaneously progress patient care while preserving a sense of learner autonomy. Rather than seeing a patient, reporting back, and “rinse-and-repeat,” acting as the lead for their patient’s care, from arrival to disposition, can convey to the learner that their contributions are valued.

Utilizing the RIME framework within the context of the ED may offer a structured approach for educators to assess students’ skills ( Ander *et al*., 2012). The RIME framework, which stands for “Reporter-Interpreter-Manager-Educator,” enables educators to observe students as they progress from basic reporting of patient information to more advanced tasks like interpretation, management, and educational roles ( Pangaro, 1999). This structured observation allows educators to pinpoint students’ strengths and areas for improvement at each stage of the clinical encounter. By employing the RIME framework, supervising clinician educators can provide students with targeted and constructive feedback that aligns with their developmental level and foster a more tailored and effective learning experience in the ED.

## Tip 4: Maximize the patient encounter

Learning opportunities abound in the ED. While it can be difficult to balance teaching and patient care during busier shifts, one way to mitigate concerns without compromising patient flow is to maximize each patient encounter. Third-year students lack the clinical expertise to fully treat patients rapidly and require more time to process information, especially early in their third year of training. Priming the student beforehand, however, by setting achievable goals ( Natesan *et al*., 2020) and providing a brief, one-minute discussion prior to patient evaluation ( Aldeen & Gisondi, 2006) can provide the learner with direction and alleviate educator uncertainty. Setting a time limit for the encounter allows the learner to focus on the more pertinent aspects of the history and examination without compromising departmental flow ( Aldeen & Gisondi, 2006).

Categorizing illnesses based on distinguishing features can aid clinicians in recognizing disease patterns and apply the correct diagnostic or treatment modality despite the vast number of possible illnesses and their often overlapping signs or symptoms. These categories, or “illness scripts,” can be rapidly and readily applied by the experienced clinician to accurately treat the patient in a time-saving, non-analytic fashion ( Rencic, 2011). Students new to the clinical environment may not be familiar with applying illness scripts to patients in their care ( Rencic, 2011). Making a conscious effort to discuss illness scripts for each item on the differential diagnosis can aid the student in comparing and contrasting different disease processes and prepare them to identify and apply them over the course of their training. The practice of interleaving, or intentionally assigning different patients with similar complaints, can also enhance learning by permitting the learner to appreciate the similarities and differences between semi-related presentations ( Natesan *et al*., 2020).

## Tip 5: Emphasize “extra-clinical” skills

Learning in the ED is not solely restricted to clinical knowledge and content. EM offers a wide variety of skills, including interprofessional collaboration, communication with patients and family members, synthesis of information, and critical decision-making ( Coates, 2004). Effective collaboration with ED nursing and ancillary staff is essential for each individual patient’s care and department throughput as a whole. Having the student act as the primary contact for communication between the physician care team and nursing staff provides the learner with a sense of autonomy, while developing critical teamwork skills. Skillful communication with patients and their family members to elicit patient preferences and provide updates regarding care plans has been identified by both attendings and students as one of the main learning needs, and is well aligned with Accreditation Council for Graduate Medical Education (ACGME) core competencies for graduating medical students ( Pusic *et al*., 2016). Communication and professionalism represent soft skills that students can develop by calling consultants, which also supplements their clinical knowledge while simultaneously assisting the care team during periods of high patient volume.

## Tip 6: Employ a variety of teaching techniques

Even among third-year students, clinical competence and knowledge will vary from student to student and by time of year. A one-size-fits-all approach to teaching is unlikely to provide educational benefit equitably across learners, and may fail to capitalize on the plethora of teachable moments available on a particular shift. Instead, best practices for feedback suggest employing a variety of teaching techniques depending on the particular learner and clinical situation ( Natesan *et al*., 2023). A number of different teaching approaches for bedside teaching have been described, including the One-Minute Preceptor, SNAPPS (Summarize, Narrow, Analyze, Probe, Plan, Select), and ED STAT (Emergency Department Strategies for Teaching Any Time) ( Natesan *et al*., 2020).

 The One-Minute Preceptor involves: 1) getting a commitment from the learner as to what they think is going on, 2) probing the learner for supporting evidence, 3) teaching general rules applicable to the case, 4) reinforcing what was done correctly, and 5) correcting learner mistakes ( Chinai *et al*., 2018; Natesan *et al*., 2020). When practiced, this model enables EM educators to quickly assess for any knowledge gaps, provide positive feedback that reinforces learning, and provide suggestions for further growth. Prompting the student to commit to an impression and verbalize their supporting evidence has the added benefit of teaching information synthesis and clinical decision making.

SNAPPS directs the learner to Summarize the history and examination, Narrow the differential to the most likely 2-3 diagnoses, Analyze the differential with supporting or refuting evidence, Probe the preceptor with self-identified knowledge gaps, develop a Plan for the patient using the teacher and additional resources, and Select a self-directed learning activity following the clinical scenario ( Chinai *et al*., 2018). This model has the benefit of being entirely learner-driven, and can be used for more advanced students later in the academic year or towards the end of their rotation. ED STAT was specifically designed for teaching in the ED, and follows its acronym by setting Expectations, Diagnosing the learner, Setting up a question relevant to the scenario, Teaching concise and high-yield pearls, Assessing and giving feedback, and reminding educators of their status as role model with Teacher always ( Chinai *et al*., 2018). While there are a number of other teaching modalities in addition to the three described, we suggest that EM educators familiarize themselves with several techniques and tailor their teaching with a modality that is most appropriate for a particular clinical situation.

## Tip 7: Develop and utilize “mini-lectures”

In contrast to inpatient clinical settings (e.g., hospital wards), busy outpatient settings only permit a fraction of total patient care time to be spent educating learners ( Pusic *et al*., 2016). Time pressures in a busy ED may make for an intimidating or anxiety-provoking learning environment for new learners ( Tews & Hamilton, 2011), and can make education challenging for faculty. One solution is teaching through occasional clinical pearls or “mini-lectures” when time permits. Developing a repository of quick teaching scripts in advance requires some degree of preparation, but can provide the learner with specific teaching points regarding common ED presentations without presenting a cognitive burden to educators. Mini-lectures can, but need not, be created *de novo*, as a number of third-party resources exist that the educator can consult. Available diagnostic or management flowcharts can shed light on the educator’s thought process and provide the learner with avenues for continued self-directed learning ( Natesan *et al*., 2020). When the patient’s clinical care takes precedence over education (e.g., acute medical resuscitations), having teaching points prepared in advance allows the educator to prioritize care for acutely-ill patients without sacrificing opportunities for workplace-based learning ( Aldeen & Gisondi, 2006). 

## Tip 8: Advise beyond EM

Traditionally reserved for fourth-year students, EM clerkships have prepared senior medical students for entering EM upon graduation, or if they choose a different field, what to expect when called to evaluate or admit patients from the ED. Third-year rotators are often undecided as to what specialty they will ultimately enter, but this does not mean EM educators should refrain from advising students on their future careers. EM complements the core specialties, such as internal medicine, surgery, and pediatrics, while exposing learners to the more acute stages of illness and/or injury they will ultimately treat in other clinical contexts ( Tews & Hamilton, 2011). Moreover, emergency physicians seamlessly work with physicians of all specialties, and are uniquely poised to provide insights into the intersection of specific specialties and their respective management ( Tews & Hamilton, 2011). Due to the high number of patients students evaluate during the ED rotation, faculty can offer insights into clinical decision-making and patient management in preparation for residency training regardless of their intended specialty. This is particularly true for high acuity patient care (e.g., managing a medical resuscitation), as all students will be expected to respond to situations of high acuity during post-graduate training.

## Tip 9: Share the teaching responsibility with others

When working with learners of multiple levels, senior residents, when available, can assist with teaching responsibilities. In the demanding and chaotic environment of the ED, sharing teaching responsibilities between faculty and residents may be beneficial in highly demanding environments ( Tews & Hamilton, 2011) and minimizes the perceived tradeoff between maintaining a high-quality patient care and providing high-value clinical teaching. Pairing a student with a resident can be particularly helpful for students in the nascent stages of their clinical development by allowing them to hone their history and examination skills as they gain the experience required to manage ED patients independently ( Coates, 2004). When working together, students gain the advantage of working with someone closer to themselves in terms of training and experience, while residents are given the opportunity to cement their knowledge by teaching junior learners.

## Tip 10: Disseminate learning objectives to faculty and residents

Disseminating teaching objectives across all educators in the clinical department ensures consistency in education and evaluation ( Tews *et al*., 2015). This requires consensus from educational leadership on what should be taught during the clerkship. Often times, these learning objectives will be shaped by medical education objectives of the medical school curriculum. Furthermore, objectives can be linked to the six core ACGME competencies deemed necessary for all practicing physicians ( Eno *et al*., 2020). Leaders in undergraduate medical education may likely have an already established curriculum depending on the institution; however, outlining specific learning goals is moot unless these goals are explicitly shared with individual instructors. Informing attendings and residents of learning objectives enables them to provide more useful feedback to students post shift. Coates (2004) writes that “daily evaluations, although time-intensive, allow the students to modify their behavior as the rotation progresses,” which has been shown to be more valued by learners than end-of-rotation evaluations ( Chaou *et al*., 2017).

## Tip 11: Implement a dedicated teaching shift in the emergency department

Competing demands between clinical practice and teaching responsibilities can be circumvented entirely with the implementation of a dedicated teaching shift. A dedicated teaching shift is an ED shift during which the educator is solely assigned to teaching learners in the department, and is completely relieved of the responsibility of clinical care. Such a shift can be staffed by either a resident, an attending, or both if scheduling and budget permit. The integration of such a shift reframes the learning experience to prioritize bedside teaching without compromising patient care. This intervention, however, requires a significant investment in terms of faculty training and staffing ( Guth *et al*., 2020). Especially for learners who are beginning their clinical experience in the ED, removal of time pressures and patient turnover allows for the extra time required to synthesize and process information and self-identify knowledge gaps. Students can re-examine patients under the direct supervision of an educator for real-time, hands-on feedback ( Coates, 2004). Educators, in turn, can stress teachable moments and model exemplary history and physical examination skills, communication, and respect for patient privacy and autonomy ( Natesan *et al*., 2020).

A dedicated “Teach Attending” has been found to be more educationally valuable than traditional presentations to clinical attendings by both learners and educators ( Aldeen & Gisondi, 2006). As previously described, providing on-shift feedback is challenged by feeling too busy on shift. Teaching shifts can untether the business of clinical work and the need for high-quality, bedside teaching ( Chaou *et al*., 2017). Another study found that the addition of a “Teach Attending” to the 3 ^rd^ year curriculum resulted in increased student evaluation scores, both for bedside teaching and the EM rotation overall ( Guth *et al*., 2020).

## Tip 12: Establish continuity with learners

Adding to the clinical unpredictability of the ED is the shift variation that results when learners are paired with different clinical teams and different clinical preceptors and educators over the course of their clerkship. Both teachers and learners need to repeatedly acclimate to one other on a daily basis, rather than being paired for longer period of time (i.e., what typically takes places during inpatient clinical learning experiences) ( Chaou *et al*., 2017). In resident education, learners benefit from this perceived drawback by being exposed to multiple clinical management styles and developing their own clinical repertoire for the practice of medicine, and working with the same providers over years of residency training.

In contrast, medical students rotate in the ED only for weeks at a time and may find it intimidating to frequently adjust to new instructors. When examining clinical uncertainty, a multi-center survey demonstrated that 30% of teachers reported difficulty adapting to different learners daily, and 50% of learners reported difficulty adapting to different teachers ( Chaou *et al*., 2019). This same survey found that both context and relationship between learner and teacher are important for providing and receiving feedback. Overcoming concerns of adaptability can be managed by scheduling students with a constant set of attendings, such that familiarity can allow for trust and stronger teacher-learner relationship. This intervention also requires a significant commitment from faculty and department leadership, but has the potential to enhance learning by promoting stronger educational alliances.

## Conclusion

The ED provides a wide array of learning opportunities for third-year medical students as they embark on their journey towards physicianhood. The variety of ED patient presentations can complement many fields, and an emphasis on extra-clinical skills will develop universal, core competencies required of all newly-minted physicians. Teaching in the busy, unpredictable clinical environment of the ED is certainly not without its challenges; but the tips outlined above can guide educators to ensure an enriching clinical experience for undifferentiated third-year medical students who may not have yet crystallized their decision to purse a specific specialty. By tailoring what and how they teach on shift, EM educators can build on existing knowledge and expand students’ toolkit in ways that will benefit them in residency and beyond, regardless of the specialty they ultimately choose.

## Data Availability

No data are associated with this article.
