# Supplementary material for: Practical tips for teaching the undifferentiated medical student in the emergency department
Source: MedEdPublish (2016). 2025 Jan 28;13:217. Originally published 2023 Oct 18. [Version 2] doi: 10.12688/mep.19776.2 (PMC11301134; doi:10.12688/mep.19776.2)
Supplement: Peer review history

# Reviewer report: Reviewer response for version 2

**DOI:** 10.21956/mep.22381.r41014

**Published:** 2025-01-30

**Authors:** Alexander Torres

**Affiliations:** 1 Medical Education, Nova Southeastern University Dr Kiran C Patel College of Allopathic Medicine (Ringgold ID: 503842), Fort Lauderdale, Florida, USA

No further comments. The updates made by the authors are well received and enhance the work.

If evidence from practice is presented, are all the underlying source data available to ensure full reproducibility?

Not applicable

Is the topic of the practical tips discussed accurately in the context of the current literature

Yes

Are all factual statements correct and adequately supported by citations?

Yes

Are the conclusions drawn balanced and justified on the basis of the presented arguments?

Yes

Are arguments sufficiently supported by evidence from the published literature and/or the authors’ practice?

Yes

Reviewer Expertise:

I am an emergency physician with additional subspecialty training in emergency medical services who currently serves as a full-time Physician Educator in undergraduate medical education at an LCME-accredited medical school. In my role as an Assistant Professor/Physician Educator, I am heavily involved in all aspects of the UME curriculum and focus on improving teaching to medical students. Additionally, I work clinically as an emergency physician at a large academic medical center where I teach students and residents at all levels of training.

I confirm that I have read this submission and believe that I have an appropriate level of expertise to confirm that it is of an acceptable scientific standard.

# Reviewer report: Reviewer response for version 1

**DOI:** 10.21956/mep.21190.r37701

**Published:** 2024-08-05

**Authors:** Alexander Torres

**Affiliations:** 1 Medical Education, Nova Southeastern University Dr Kiran C Patel College of Allopathic Medicine (Ringgold ID: 503842), Fort Lauderdale, Florida, USA

I am an emergency physician with additional subspecialty training in emergency medical services who has practiced in all types of settings (community, academic, and military). My current practice is divided into mostly teaching undergraduate medical education (UME) in the pre-clinical phases (accounts for approximately 85% of my time) and teaching medical students and residents in the clinical setting as part of an emergency medicine residency program (15% of my time). Given my practice history and current educational role, I am deeply familiar with UME and graduate medical education (GME) in the United States (US), and therefore frame my review from the perspective of medical education in the US.

**Summary**: The authors have identified critical considerations regarding teaching third-year medical students rotating in the emergency department, and importantly address the variability in foundational and clinical knowledge compared to their fourth-year counterparts, and even the variability within their own third-year cohorts depending upon in which part of the year the rotation occurs. While there is discussion regarding the “undifferentiated” medical student, the article addresses overall theory and tips for teaching emergency medicine to students given the unique practice environment. The article is well written and has an overall good flow regarding the background of emergency medicine UME education, evidence-based historical facts and suggested resources, as well as tips for educators that can be used regardless of the acuity and pace of any shift. The authors propose both educator-based and administrative-based tips, some of which can be more easily implemented compared to others. Ultimately, the authors provide various suggestions for keeping teaching focused on the learner and providing ideas on how to adapt teaching experiences to the situation at hand.

**Strengths**:

1. The authors address the complexities of emergency medicine practice environment while also acknowledging the positive aspects, such as breadth of pathology, acuity, volume, and interprofessional collaboration.
2. At multiple points in the article, the authors emphasize the need to keep teaching focused on the learner’s needs and experiences. A significant strength of this article is the evidence-based suggestions for teaching tools (One-Minute Preceptor, SNAPPS, and ED STAT) that can be utilized as well as which are better suited for more advanced learners. Additionally, the RIME framework allows for a structured approach that can be applied by all educators and students to allow for standardization and helps to set expectations early on.
3. The 12 tips presented keep focus on the student experience while addressing multiple factors that must be considered in emergency medicine teaching (time limitations, unpredictability, administration, etc.). The authors provide recent and relevant citations for each tip presented which strengthens the validity and applicability of these tips.  

**Limitations**:

1. The abstract and introduction address that more schools are moving emergency medicine clerkships into the third-year; however, a critical factor to consider regarding third-year medical students is the variability of clinical knowledge (and ability to apply their pre-clerkship learning to clinical situations) depending upon which point in the third-year that their emergency medicine rotation occurs. This point is addressed later in the article (in ‘Tip 1’) but it should be addressed in the Introduction as the underlying concept is applicable to all the tips presented.
2. The authors discuss the need for interprofessional collaboration (Tip 5) without addressing the challenges associated with these collaborations. The authors recommend having the student act as the primary contact for communication between the physician care team and nursing staff, which at the core is a great way to engage and empower the student, however, practically, this requires buy-in from nursing and ancillary staff. The authors should address the need to have nursing (and ancillary service) administration be engaged in this part of interprofessional collaboration as part of the medical student experience. Additionally, when suggesting that the student be the primary contact, it is imperative to address times in which the student is not the appropriate primary contact (i.e. critical lab values, time-sensitive medical conditions/changes in condition, etc.), and how this might impact the student’s experience, sense of autonomy, and sense of inclusion as part of the healthcare team. The authors accurately describe the unique experiences of emergency physicians with regards to communicating with patients and families, other physicians/consultants, ancillary staff, and multiple other members of the healthcare team which distinctively positions emergency physicians to provide well-thought out recommendations regarding medical specialty choices and ad-hoc advising for undifferentiated medical students.  

**Additional Thoughts**:

 Given the variability and breadth of potential student experiences in the emergency department and the fact that there is no guarantee regarding the types and volume of exposure to specific pathologies, the use of orientation materials and asynchronous learning for certain skills and conditions can be helpful adjuncts for medical students on an emergency medicine rotation. Since emergency medicine and critical care rotations have many similarities, the authors should consider reference to orientation and adjunct educational materials similar to what has been done in the critical care education setting. A recent article published in October 2023 in ATS Scholar by Maria C. Cañizares-Otero et al. addresses how the implementation of an orientation manual in an intensive care unit rotation can enhance the educational experience for trainees.

If evidence from practice is presented, are all the underlying source data available to ensure full reproducibility?

Not applicable

Is the topic of the practical tips discussed accurately in the context of the current literature

Yes

Are all factual statements correct and adequately supported by citations?

Yes

Are the conclusions drawn balanced and justified on the basis of the presented arguments?

Yes

Are arguments sufficiently supported by evidence from the published literature and/or the authors’ practice?

Yes

Reviewer Expertise:

I am an emergency physician with additional subspecialty training in emergency medical services who currently serves as a full-time Physician Educator in undergraduate medical education at an LCME-accredited medical school. In my role as an Assistant Professor/Physician Educator, I am heavily involved in all aspects of the UME curriculum and focus on improving teaching to medical students. Additionally, I work clinically as an emergency physician at a large academic medical center where I teach students and residents at all levels of training.

I confirm that I have read this submission and believe that I have an appropriate level of expertise to confirm that it is of an acceptable scientific standard.

## References

[1] : Implementation and Evaluation of a Trainee Orientation Manual in an Intensive Care Unit Rotation. *ATS Sch* .2024;5(1) : 10.34197/ats-scholar.2023-0007OC 109-121 10.34197/ats-scholar.2023-0007OC 38628303 PMC11019769

# Reviewer report: Reviewer response for version 1

**DOI:** 10.21956/mep.21190.r35089

**Published:** 2023-11-14

**Authors:** Michael Cassara

**Affiliations:** 1 Graduate Nursing and Hofstra Northwell School of Nursing and Physician Assistant Studies, Hofstra University, Hempstead, New York, USA; 2 Center for Learning and Innovation, Northwell Health, New Hyde Park, New York, USA

In full transparency, I am an emergency medicine physician trained and certified in the United States. Despite my best attempts to frame my review within a global perspective, I may describe medical education and trajectory of physician education using language and conventions consistent with my American experience. Please disregard any perceived ethnocentrism as a result; it is unintended and unintentional.  

**Summary:** The authors present 12 theory-informed, evidence-supported tips for educators of "undifferentiated" medical students situated with the learning environments of the emergency department (ED) prior to their final (fourth) year of undergraduate medical education. The authors define "undifferentiated" medical students as medical students who have advanced beyond pre-clinical (university-centered) education to engage in instruction that is predominantly patient-facing, hospital-centered, and highly clinical in nature. The authors also contrast "undifferentiated" medical students with senior (fourth) year students in that the former represent a cohort of learners who have great variability in desired future specialty and career plans. In the manuscript, the authors posit that, as the educational goals for these learners are inherently different from those learners with specific interest in careers in emergency medicine, educators precepting these more junior learners would benefit from a comprehensive review of literature-informed best practices and guiding principles, especially those more accustomed to precepting learners at later developmental stages. The authors propose eight tips that are learner-centered, and four additional tips that center on systemic (departmental) considerations related to the learning environment/learning climate. 

 Overall, I found this manuscript to be an enjoyable read that will benefit clinician educators working in academic emergency departments where these undifferentiated medical students may rotate.

**Strengths:**

1. The authors effectively use Watling and Lingard's (2015) "problem-gap-hook" heuristic to frame the beginning of the manuscript. Thank you.
2. The authors appropriately highlight several strengths of the emergency department as rich learning environments for undifferentiated medical students, all of which I agree with. I found the authors evenly distributed these strengths among the 12 tips. Thank you.
3. The authors have curated 12 tips that I believe are highly defensible for inclusion in a manuscript of this type (authors provide sufficient "validity evidence" supporting their inclusion in a "12 Tips" paper, in my opinion). The authors provide factually correct statements in support of the 12 tips they selected. and, for most, adequately support these statements using recent citations by well recognized authors. Thank you. 
4. The authors highlight several best practices, theories, and guiding principles of medical education that align well with the instruction of "undifferentiated" medical student learners in a complex and chaotic milieu that is the emergency department. I feel every tip - from Tip 1 to Tip 12 - contains one or more examples I would categorize as "must know" strategies and concepts, including but not limited to: One-Minute Preceptor, SNAPPS, ED STAT, RIME, cognitive load theory, microteaching, and Socratic questioning. Thank you.

**Limitations:**

1. Despite the authors' overall effective use of the aforementioned "problem-gap-hook" heuristic in the Introduction, the authors' start the Introduction with a first sentence that does not make sense and requires revision. In addition, the authors could enhance the Introduction by providing data (if available) that explicitly demonstrate the increasing prevalence of institutions moving emergency medicine clerkships from the final (fourth) year into earlier portions of the clinical period of training. I ask the authors to consider implementation of a revision based on this recommendation.
2. I feel the authors could have used this manuscript as an opportunity to more deeply describe how educators within the emergency department milieu could provide undifferentiated medical students with unique instruction in the principles of interprofessional collaborative practice, team science, and systems-based practice. I believe these principles are those which undifferentiated medical students would benefit from learning while on rotation in the emergency department. Almost every aspect of emergency department patient care requires members to serve in teams where roles are dynamic, clear communication is required, situational awareness is key, and collaboration among team members, family members, and patients is critical. The emergency department represents an excellent environment in which to highlight this knowledge domain and these competencies. Highlighting these concepts, along with the interprofessional approach to patient care that often exists in the emergency department, could have been effectively introduced and applied to recommendations made by the authors in the Tip 5 and Tip 9 sections of the manuscript. I ask the authors to consider implementation of a revision based on this recommendation. 

**Other thoughts:**

1. One of the methods I had hoped the authors would include as part of the manuscript is the FED (feedback-encouragement-direction) model as proposed by Bell (2007 ^1^) as it is learner-centered and quickly implemented in the clinical environment. Anecdotally, educators at our institution integrate learner self-assessment at the beginning of this method to form SFED (self-assessment-feedback-encouragement-direction), a model similar to Ask-Tell-Ask. For the authors' consideration.
2. Tip 10 - dissemination of learning objectives related to undifferentiated medical student emergency department rotators to emergency department faculty and residents - could be augmented with the use of a learning management system. With the ubiquitous availability of free, commercially available online LMS platforms (e.g., Google Classroom [Alphabet, Inc., Mountain View, CA] or equivalent), and given the likelihood that many emergency medicine faculty and residents do not have instructor access to one or more medical schools LMSs, I thought this would have been a useful recommendation to include alongside the other statements. For the authors' consideration.

If evidence from practice is presented, are all the underlying source data available to ensure full reproducibility?

Not applicable

Is the topic of the practical tips discussed accurately in the context of the current literature

Yes

Are all factual statements correct and adequately supported by citations?

Yes

Are the conclusions drawn balanced and justified on the basis of the presented arguments?

Yes

Are arguments sufficiently supported by evidence from the published literature and/or the authors’ practice?

Yes

Reviewer Expertise:

Health professions education, Interprofessional education, Medical education, Healthcare simulation, Education research, Education scholarship, Systematic reviews

I confirm that I have read this submission and believe that I have an appropriate level of expertise to confirm that it is of an acceptable scientific standard.

## References

[1] : Encouragement: giving "heart" to our learners in a competency-based education model. *Fam Med* .2007;39(1) :13-5 17186439
